# Tandem Mass Tags Quantitative Proteome Identification and Function Analysis of ABC Transporters in *Neofusicoccum parvum*

**DOI:** 10.3390/ijms23179908

**Published:** 2022-08-31

**Authors:** Jie Chen, Shan Han, Shujiang Li, Hanmingyue Zhu, Shuying Li, Junjie Yan, Tianhui Zhu

**Affiliations:** 1 College of Forestry, Sichuan Agricultural University, Chengdu 611130, China; 2 National Forestry and Grassland Administration Key Laboratory of Forest Resources Conservation and Ecological Safety on the Upper Reaches of the Yangtze River, Chengdu 611130, China; 3 Institute of Urban Agriculture, Chinese Academy of Agricultural Sciences, Chengdu 610213, China

**Keywords:** *NpABC2*, ATP-binding cassette transporter, TMT protein quantification, *Neofusicoccum parvum*, gene knockout

## Abstract

*Neofusicoccum parvum* can cause twig blight of the walnut (*Juglans spp.*), resulting in great economic losses and ecological damage. We performed proteomic tandem mass tags (TMT) quantification of two *Neofusicoccum parvum* strains with different substrates, BH01 in walnut substrate (SW) and sterile water (SK), and BH03 in walnut substrate (WW) and sterile water (WK), in order to identify differentially expressed proteins. We identified 998, 95, and 489 differentially expressed proteins (DEPs) between the SK vs. WK, SW vs. SK, and WW vs. WK comparison groups, respectively. A phylogenetic analysis was performed to classify the ABC transporter proteins annotated in the TMT protein quantification into eight groups. Physicochemical and structural analyses of the 24 ATP-binding cassette (ABC) transporter proteins revealed that 14 of them had transmembrane structures. To elucidate the functions of these transmembrane proteins, we determined the relative expression levels of ABC transporter genes in strains cultured in sodium chloride, hydrogen peroxide, copper sulfate, and carbendazim mediums, in comparison with pure medium; analysis revealed differential upregulation. To verify the expression results, we knocked out the *NpABC2* gene and compared the wild-type and knockout mutant strains. The knockout mutant strains exhibited a higher sensitivity to antifungal drugs. Furthermore, the virulence of the knockout mutant strains was significantly lower than the wild-type strains, thus implying that *NpABC2* plays a role in the drug resistance of *N. parvum* and affects its virulence.

## 1. Introduction

ABC transporter proteins are commonly found in all living organisms [1]. ABC transporters are distributed on cell membranes and organelle membranes, and their main function is to transport various substrates (sugars, amino acids, metal ions, peptides, proteins, hydrophobic compounds, and their metabolites) against a chemical gradient driven by ATP hydrolysis [2]. Depending on the direction of substrate transport, these transporters can be divided into importing and exporting types; yet, in almost all eukaryotes, ABC family members function act as exporters [3]. The canonical structure of an ABC transporter is organized into four domains, two nucleotide-binding domains (NBDs) and two transmembrane domains (TMDs). The TMDs contain the substrate-binding site and act as the transmembrane bilayer transport channel; the NBDs is the region responsible for ATP binding and hydrolysis.

The NBD is a hallmark of the ABC transporter family because of its conserved sequence and the presence of several conserved motifs [4]. The NBDs within and between bacterial and eukaryotic exporters exhibit similar three-dimensional folding and conserved mechanisms related to energy coupling [4]. Each NBD can be divided into two subdomains, a catalytic core domain and an α-helical domain. The catalytic core domain contains conserved motifs for the binding and hydrolysis of ATP; the Walker A motif (P-loop) interacts with phosphate groups of the nucleotide while the Walker B motif contains a glutamate residue that acts as a general base to activate a water molecule for nucleophilic attack at the γ-phosphate of ATP. The α-helical domain contains the motif LSGGQ; this is known as the ABC-signature motif (or C-loop) that is involved in nucleotide binding [5,6,7]. The NBDs are arranged ‘head-to-tail’ and form the full composite site for ATP-binding and hydrolysis upon dimerization via interactions with the D-loop; the ATP-binding site is formed from the Walker A and Walker B motifs of one subunit and the Q-loop of the other subunit, so that two ATP molecules can bind and hydrolyze [3]. In the nucleotide-free state, the NBDs are separated and exhibit an open conformation; however, upon ATP binding, they come close together to sandwich the ATP molecules in a closed conformation [3,8,9]. Each eukaryotic TMD has six transmembrane α-helices which form a transmembrane pore that is either accessible from the cytoplasm or the outside of the cell [10,11,12]. Unlike the NBDs, the TMDs generally exhibit no significant sequence conservation but share a similar topology within the transporter class [4]. Full transporters (FT) feature two TMD and two NBD domains, while half transporters (HT) feature one TMD and one NBD domain.

Some ABC transporter genes can influence the virulence of phytopathogenic fungi. In total, 50 genes have been found to encode the ABC transporter in the genome of *Magnaporthe grisea* [13]. The genes ABC1–7 have been shown to be involved in many functional roles, including the formation of early infestation forms during host infestation by the pathogenic fungus, defense against cytotoxic substances produced by the attached intracellular host and in the oxidative stress response of the host, in multidrug resistance, the virulence of *M. grisea*, and in abiotic stress tolerance and conidia formation [13,14,15,16,17]. An analysis of eleven ABC transporter genes (*BcatrA–K*) of *Botrytis cinerea* showed that *BcatrB*, *BcatrD,* and *BcatrK* are determinants of sensitivity to fungicides [18,19,20,21,22,23].

*Neofusicoccum parvum* is an important pathogen that causes twig blight and affects a wide range of hosts, including a large number of fruit trees [24,25,26,27,28], nut trees [29,30], and oil trees [31]. This pathogen is responsible for walnut branch blight disease in Sichuan, China [32]; however, common measures are ineffective and cannot contain the development of this disease [33].

In this study, we performed a quantitative analysis of the *N. parvum* proteome between pure and walnut-supplemented potato dextrose agar (PDA) medium as a growth substrate. We revealed a large number of differentially expressed proteins and hypothesized that the presence of abundant ABC transporter proteins might provide *N. parvum* with a strong drug resistance and infestation amplification ability. We also performed a phylogenetic analysis of the ABC transporters and analyzed their properties and structures. To explore their functional roles, we determined the expression of the genes encoding these proteins by quantitative real-time polymerase chain reaction (PCR). Finally, we verified our data by knocking out the *NpABC2* gene and assaying the stress resistance, drug resistance, and virulence of the knockout mutant strains, thus providing a reference for further functional studies of ABC transporters in fungi.

## 2. Results

### 2.1. Quantitative Proteomic Analysis

#### 2.1.1. Proteomic Data Quality Control and Identification

The total number of secondary spectra obtained after searching and filtering the data was 419,577, the number of valid spectra was 93,734, the number of peptides identified was 44,599, the number of proteins identified was 5017, and the total number of quantifiable proteins finally obtained was 5013. The quality control report for the proteomics data is shown in Figure S1. The proteomics data obtained from mass spectrometry have been deposited to the ProteomeXchange Consortium (http://proteomecentral.proteomexchange.org, accessed on 3 June 2022) via the iProX partner repository [34] with the dataset identifier PXD034277.

#### 2.1.2. Protein Function Annotation

**Figure 1:**
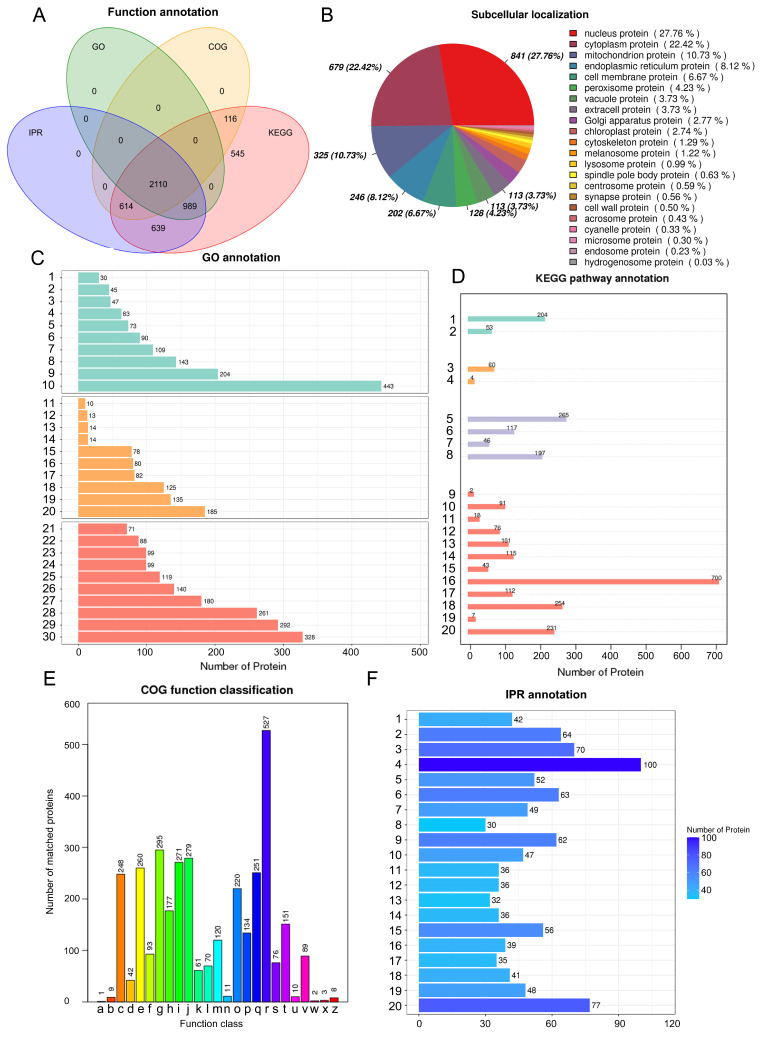
Functional annotation of proteins. (**A**): Venn diagram showing the annotation results from the GO, COG, IPR, and KEGG databases. (**B**): Pie charts showing protein subcellular localization. (**C**): Histogram showing GO annotation results. Green columns represent biological process (1–10), 1: small GTPase mediated signal transduction; 2: intracellular protein transport; 3: transport; 4: protein phosphorylation; 5: proteolysis; 6: translation; 7: carbohydrate metabolic process; 8: transmembrane transport; 9: metabolic process; 10: oxidation–reduction process. Yellow columns represent cellular components (11–20), 11: eukaryotic translation initiation factor 3 complex; 12: mitochondrion; 13: proteasome core complex; 14: mediator complex; 15: cytoplasm; 16: intracellular; 17: ribosome; 18: nucleus; 19: membrane; 20: integral component of membrane. Orange columns represent molecular functions (21–30), 21: metal ion binding; 22: structural constituent of ribosome; 23: DNA binding; 24: hydrolase activity; 25: nucleic acid binding; 26: zinc ion binding; 27: catalytic activity; 28: oxidoreductase activity; 29: ATP binding; 30: protein binding. (**D**): Histogram showing KEGG annotation results: cellular processes (1–2), 1: transport and catabolism; 2: cell growth and death; environmental information processing (3–4), 3: signal transduction; 4: membrane transport; genetic information processing (5–8), 5: translation; 6: transcription; 7: replication and repair; 8: folding, sorting, and degradation; metabolism (9–20), 9: xenobiotics biodegradation and metabolism; 10: nucleotide metabolism; 11: metabolism of terpenoids and polyketides; 12: metabolism of other amino acids; 13: metabolism of cofactors and vitamins; 14: lipid metabolism; 15: glycan biosynthesis and metabolism; 16: global and overview maps; 17: energy metabolism; 18: carbohydrate metabolism; 19: biosynthesis of other secondary metabolic; 20: amino acid metabolism. (**E**): Histograms of COG-annotated results; a: RNA processing and modification; b: chromatin structure and dynamics; c: energy production and conversion; d: cell cycle control, cell division, chromosome partitioning; e: amino acid transport and metabolism; f: nucleotide transport and metabolism; g: carbohydrate transport and metabolism; h: coenzyme transport and metabolism; i: lipid transport and metabolism; j: translation, ribosomal structure, and biogenesis; k: transcription; l: replication, recombination, and repair; m: cell wall membrane/envelope biogenesis; n: cell motility; o: posttranslational modification, protein turnover, chaperones; p: inorganic ion transport and metabolism; q: secondary metabolites biosynthesis, transport, and catabolism; r: general function prediction only; s: function unknown; t: signal transduction mechanisms; u: intracellular trafficking, secretion, and vesicular transporter; v: defense mechanisms; w: extracellular structures; x: mobilome: prophages, transposons; z: cytoskeleton. (**F**): Histograms showing IPR annotation results; 1: Zn(2)-C6 fungal-type DNA-binding domain; 2: WD40-repeat-containing domain; 3: WD40 repeat; 4: short-chain dehydrogenase/reductase SDR; 5: RNA recognition motif domain; 6: protein kinase domain; 7: polyketide synthase, enoylreductase domain; 8: mitochondrial substrate/solute carrier; 9: major facilitator superfamily domain; 10: major facilitator superfamily; 11: helicase superfamily 1/2, ATP-binding domain; 12: helicase, C-terminal; 13: G-protein beta WD-40 repeat; 14: cytochrome P450, E-class, group I; 15: cytochrome P450; 16: ATPase, AAA-type, core; 17: alpha/beta hydrolase fold-1; 18: alcohol dehydrogenase, N-terminal; 19: alcohol dehydrogenase, C-terminal; 20: AAA+ ATPase domain.

The 5013 proteins identified were functionally annotated, including 3099 proteins in the Gene Ontology (GO) database, 3205 proteins in the Cluster of Orthologous Groups of Proteins (COG) database, 5013 proteins in the Kyoto Encyclopedia of Genes and Genomes (KEGG) database, and 4352 proteins in the interProScan (IPR) database (Figure 1A). A subcellular localization analysis of all proteins (Figure 1B) revealed that a total of 22 proteins exhibited a subcellular localization; the largest number of proteins annotated were nuclear proteins (27.76%), followed by cytoplasmic proteins (22.42%) and mitochondrial proteins (10.73%); the lowest number of proteins were oxidasomal proteins (0.03%). Using the COG database to annotate proteins (Figure 1C), the largest number of COGs in the resulting 26 categories of different functions were predicted to be related to general function only (527 COGs); the function of 76 genes was unknown. Some genes were closely annotated to some interesting COGs. For example, 295 genes were annotated to carbohydrate transport and metabolism, 134 genes were annotated to organic ion transport and metabolism (non-organic ion transport and metabolism), 251 genes were annotated to secondary metabolite biosynthesis, transport, and catabolism, and 151 genes were annotated to signal transduction mechanisms. KEGG pathway annotation was performed for all proteins (Figure 1D); the pathway category with the most annotated proteins was metabolism with 1750 proteins, followed by genetic information processing with 625 proteins; cellular processes and environmental information processing were annotated to 257 and 64 proteins, respectively. Annotation of the structural domains of all proteins (Figure 1E) identified structural domains related to ATP binding, as well as cytochrome P450 and the transporter protein superfamily. GO functional analysis (Figure 1F) revealed that biological process (BP) processes were annotated to terms related to signal transduction, intracellular protein transport, transmembrane transport, carbohydrate metabolism, and protein and energy metabolism. Molecular function (MF) processes were associated with terms related to metal ion binding, protein binding, hydrolase activity, and energy metabolism.

#### 2.1.3. Protein Quantification and Differential Analysis

**Figure 2:**
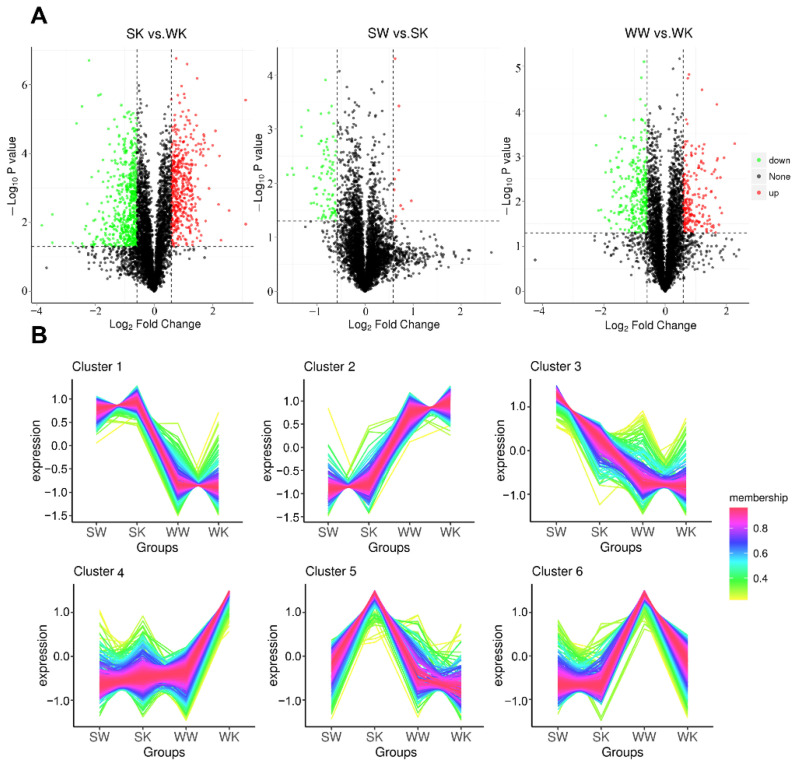
Differential protein volcano map and clustering map. (**A**): Volcano plot of differentially expressed proteins. Red dots indicate up-regulated genes and green dots indicate down-regulated genes; (**B**): C-means cluster plots of differentially expressed proteins.

The expression levels of up-regulated proteins were selected for analysis when |log2FoldChange| ≥ 1.5 and *p* ≤ 0.05. The expression levels of down-regulated proteins were analyzed when |log2FoldChange| ≤ 0.67 and *p* ≤ 0.05. For each protein, differential ploidy was taken as a logarithm with a base of 2, and the *p* value was taken as the absolute value of the logarithm with a base of 10 to generate a differential protein volcano plot for each comparison group (Figure 2A). Our analysis showed that the highest number of differentially expressed proteins when compared between the SK vs. WK group was 448 up-regulated proteins and 550 down-regulated proteins. The lowest number of differentially expressed proteins between the SW vs. SK group was 10 up-regulated proteins and 85 down-regulated proteins. In total, 211 of the differentially expressed proteins were up-regulated between the WW vs. WK group. To determine the correlation between protein expression and different experimental conditions, we performed a protein expression level clustering analysis. The expression of all samples combined was subjected to C-means clustering analysis. The correlation of categorical protein expression within and between sample groups is shown in Figure 2B. The categorical protein expression was more consistent within the sample groups; categorical protein expression of the samples between the groups showed slight differences.

#### 2.1.4. Differentially Expressed Protein Enrichment Analysis

**Figure 3:**
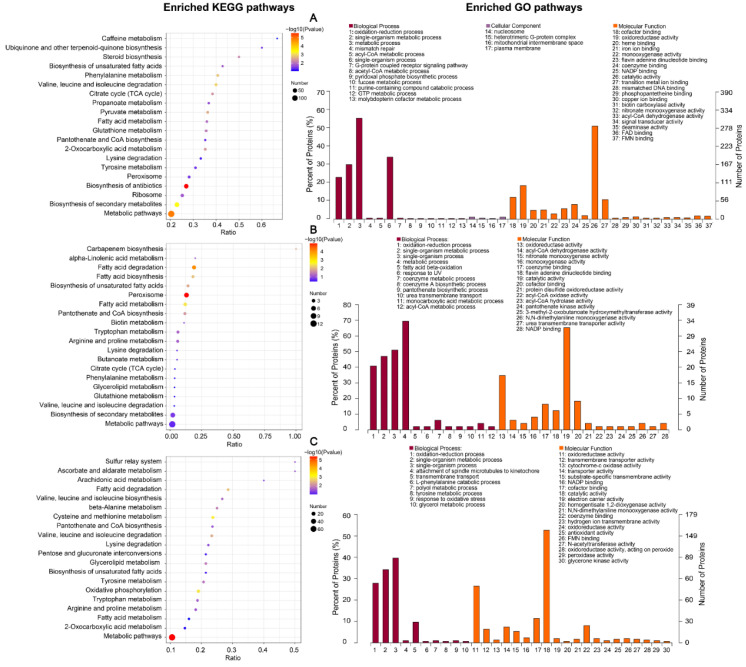
KEGG and GO enrichment analysis of DEPs. (**A**): SK vs. WK; (**B**): SW vs. SK; (**C**): WW vs. WK. The 20 most significant pathways in the GO and KEGG enrichment results from compared groups (*p* ≤ 0.05). The ratio indicates the ratio of the number of genes annotated into that GO pathway and the total number in differential genes in that group. The -log10(*p* value) indicates the significant difference of GO enrichment, indicated by different colors in the plots, and the size of the bubble indicates the number of genes in the pathway.

Data arising from KEGG and GO enrichment of the differential proteins are shown in Figure 3. The enrichment analysis for all three comparison groups showed enrichment for multiple entries, indicating that our differential factors between different comparison groups were valid. The differential factor between the SK vs. WK groups was a different strain; KEGG enrichment analysis showed enrichment in biosynthesis and degradation processes of primary and secondary metabolites, the citric acid cycle, peroxisomes, and other pathways. From this, we inferred that the differences between strains BH01 and BH03 exist mainly in their metabolic capacity, respiratory strength, and resistance to peroxides; these factors may account for some differences in their virulence. GO term enrichment also highlighted the difference between strains; enrichment was also evident in CC components in addition to BP and MF. The main enriched terms in the cellular component (CC) category included several important cell structures, including the nucleus, mitochondria, heterotrimeric G-protein complex, and plasma membrane. However, the enrichment of these protein terms was small, possibly indicating that there are subtle differences in the cytoarchitecture of the two strains. In addition, the BP fraction was mainly enriched for some energy metabolic processes such as redox processes, while the MF fraction was enriched for metal ions and terms related to energy metabolism, enzyme activity, and signal transduction.

The differential factor within the SW vs. SK groups was the presence or absence of host tissue stimulation; KEGG analysis demonstrated enrichment in carbapenem synthesis, peroxisomes, the citric acid cycle, and the synthesis and degradation of primary and secondary metabolites. The results of GO enrichment also indicated an increase in metabolism, the production of antibiotics, and the response to superoxide stress, as shown by GO enrichment analysis in which the BP and MF fractions were enriched but not the CC fraction. The main enriched pathways involved enzymatic activities related to energy metabolism.

**Figure 4:**
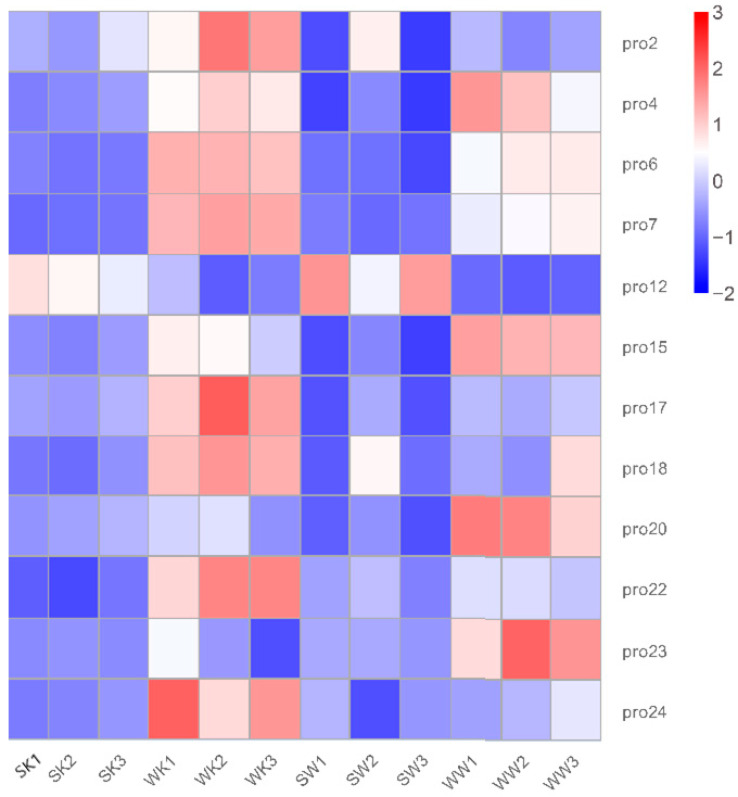
Heatmap of ABC transporters in the expression of DEPs across 12 samples. Red represents the degree of increase, while blue represents the degree of decrease.

The differential factor between the WW vs. WK groups was also the presence or absence of host tissue stimulation; KEGG analysis revealed enrichment in primary metabolites and stimulated metabolite synthesis and degradation processes. GO analysis revealed enrichment in the BP fraction for pathways associated with transmembrane transport, the response to oxidative stress, and primary metabolic processes. The MF fraction was enriched in transmembrane transport protein activity, energy metabolic processes, and antioxidant activity. This may indicate that the BH03 strain undergoes increased levels of active transport after stimulation by host tissues and produces a response to oxidative stress. We noted that differences existed among the groups, including in oxidative stress and transmembrane transport. We observed that differences such as oxidative stress and transmembrane transport existed among the compared groups; therefore, we analyzed the expression of ABC transporters among the differentially expressed proteins (Figure 4). We found that half of the 24 annotated ABC transporters were differentially expressed proteins. Thus, we considered ABC transporter genes to be comparatively important differentially expressed proteins. These ABC transporters were expressed at similar levels within groups but with relative differences between groups. Under the same conditions, the expression of 11 proteins, (except for pro12) was higher in strain BH03 than in strain BH01; therefore, we chose to perform gene knockout experiments on strain BH03.

#### 2.1.5. Subcellular Localization Analysis of DEPs

**Figure 5:**
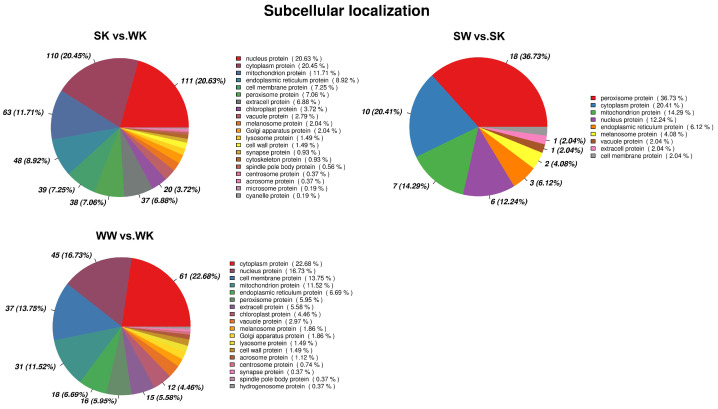
Pie chart showing the subcellular localization of differential proteins.

Subcellular localization analysis was performed for proteins showing differential expression between each comparison group (Figure 5). We found that the top three differential proteins, with the highest extent of subcellular localization, were cytosolic, cytoplasmic, and mitochondrial proteins in the SK vs. WK group; peroxisomal, cytoplasmic, and mitochondrial proteins in the SW vs. SK group, and cytoplasmic, cytosolic, and cell membrane proteins in the WW vs. WK group. This was also consistent with the results arising from differential protein enrichment analysis. Although the most enriched sites of differential proteins differed between the three comparison groups, for all three comparison groups, the top nine subcellular locations were in the nucleus, cytoplasm, mitochondria, endoplasmic reticulum, cell membrane, peroxisomes, the extracellular region, vacuoles, and melanosomes, possibly indicating that the proteins in the three different comparison groups did not differ extensively. Only differential proteins in the SK vs. WK and WW vs. WK groups showed differential proteins localized to the chloroplasts, potentially indicating that there were differences in the ability of different strains to attack the host chloroplast.

### 2.2. ABC Transporter Protein Analysis

#### Phylogenetic and Structural Analysis of ABC Transporter Proteins

**Figure 6:**
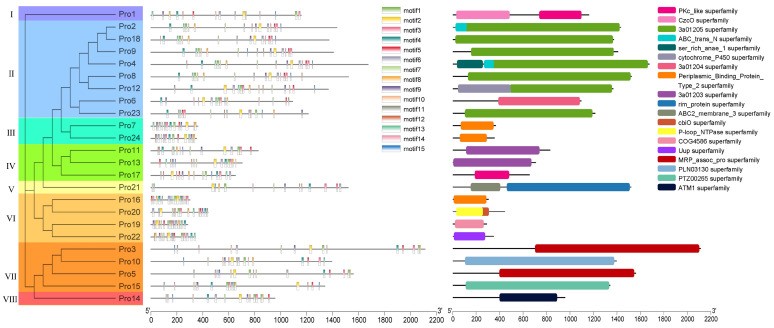
Analysis of ABC transporter proteins.

In total, 24 ABC transporter proteins (code: pro1–24) were annotated in the functional annotation results arising from proteome quantification. Phylogenetic analysis and conserved structural domain motif and domain analysis (Figure 6) showed that these proteins were divided into eight groups (Ⅰ–Ⅷ) and that the structural domains in each group were similar. From this, we hypothesize that it is possible that only 24 ABC transporter proteins were expressed under the present experimental treatments; the other 27 might need to be induced to be expressed under specific conditions, with all ABC transporter proteins in the two groups requiring specific conditions to be expressed.

**Figure 7:**
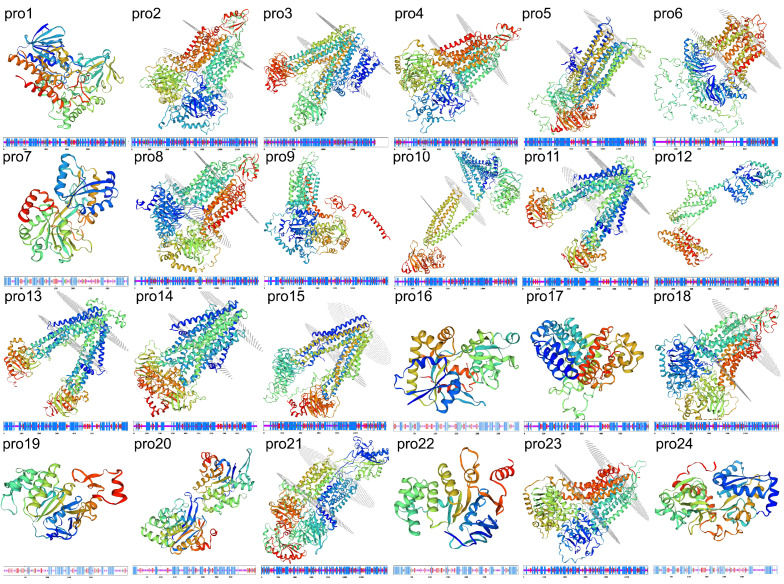
Homology modeling and secondary structures of 24 ABC transporter proteins.

To infer the functions of the 24 ABC transporter proteins, a series of predictive analyses were performed. Firstly, we analyzed their physicochemical properties (Table S1). Hydrophilic and hydrophobic analyses of the proteins are shown in Figure S2. To understand which of these proteins are secreted proteins, we predicted their signal peptides (Figure S3) and found that only 7 proteins showed signal peptides: pro5, pro6, pro7, pro12, pro14, pro21, and pro24. Secondary and tertiary structure modeling analysis (Figure 7) showed that there were 14 transmembrane proteins: pro2, pro3, pro4, pro5, pro6, pro8, pro10, pro11, pro13, pro14, pro15, pro18, pro21, and pro23; the other 10 proteins were non-transmembrane proteins.

Subcellular localization analysis revealed nine cell membrane proteins (pro2, pro4, pro8, pro9, pro12, pro14, pro15, pro18, and pro23), two cytoplasm proteins (pro19 and pro22), three vesicle and vacuole proteins (pro3, pro5, and pro10), three mitochondrial proteins (pro1, pro16, and pro20), two peroxisome proteins (pro1 and pro13), one Golgi protein (pro21), and one endoplasmic reticulum protein (pro6); the localization of the other three proteins remains unknown.

### 2.3. Analysis of Protein-Protein Interaction (PPI) Network in ABC Transporters

**Figure 8:**
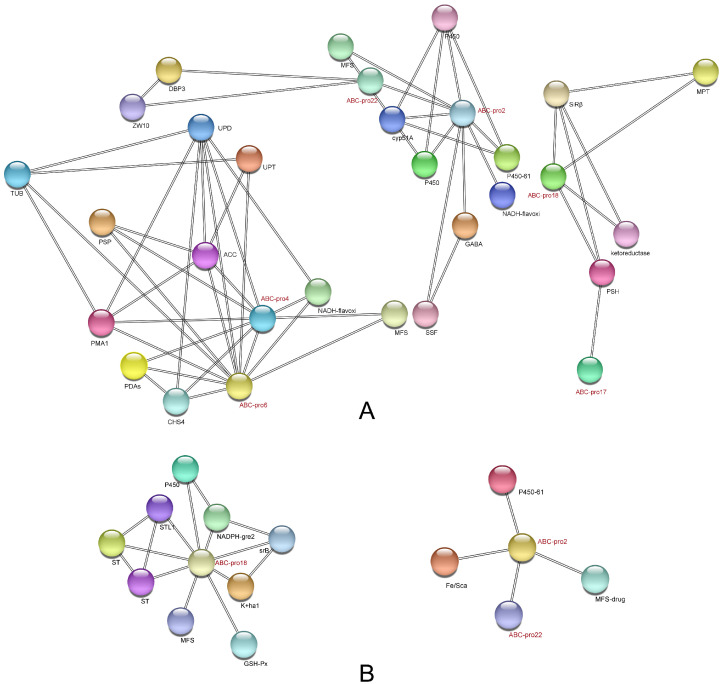
PPI network of DEPs about ABC transporters across three comparison groups. (**A**) SK vs. WK, (**B**) WW vs. WK. Only the first neighbors associated with ABC transporters are shown.

PPI network analysis of the DEPs of the three comparison groups revealed that no interacting ABC transporters were present in the SW vs. SK group, but they were present in the other two groups. In the comparison of group SK vs. WK (Figure 8A), several proteins were associated with pro4 and pro6, including tubulin beta chain, acetyl carboxylase protein, chitin synthase 4 protein, flavin oxidoreductase NADH oxidase protein, uroporphyrinogen decarboxylase protein, uracil phosphoribosyl transferase protein, patatin-like serine protein, plasma membrane H+/− ATPase protein, major facilitator superfamily transporter protein, and polysaccharide deacetylase family protein. In addition, enrichment was observed in the following GO functions: nucleotide binding (KW-0547), ribonucleotide binding (GO:0032553), catalytic activity (GO:0003824), purine ribonucleotide binding (GO:0032555), purine ribonucleotide triphosphate binding (GO:0035639), anion binding (GO:0043168). Pro2 and pro22 interact with each other and with other ten proteins, including four cytochrome p450 proteins, MFS multidrug transporter protein, solute symporter transporter protein, ATP-dependent RNA helicase dbp3 protein, NADH-dependent flavin oxidoreductase protein, centromere kinetochore protein zw10 protein, and GABA permease protein. Pro18 and pro17 interact together with patatin-like serine hydrolase protein, and pro18 interacts with another three proteins, including molybdopterin binding protein, ketoreductase protein, and sulfite reductase protein.

In the comparison group WW vs. WK (Figure 8B), nine proteins were associated with pro18; they are three sugar transporter family proteins, k+ homeostasis protein, and MFS transporter protein. Enrichment was observed in the following GO functions: proton transmembrane transporter activity (GO:0015078), active transmembrane transporter activity (GO:0022804), transmembrane transporter activity (GO:0022857), transport (KW-0813), secondary active transmembrane transporter activity (GO:0015291), active ion transmembrane transporter activity (GO:0022853) and carbohydrate: proton symporter activity (GO:0005351). Pro2 and pro22 interact with each other and with MFS multidrug transporter protein, cytochrome p450 protein, and iron sulfur cluster assembly protein.

Meanwhile, the results of PPI showed that three associated ABC transporter proteins were present in both groups, which were pro2, pro18, and pro22. This suggests that these three ABC transporters may be associated with the virulence of *N. parvum*. ABC1 encodes pro22 and five other ABC transporter proteins, ABC2 encodes pro18 and pro9, ABC13 encodes pro2, and ABC1 has been shown to be involved in virulence and peroxide resistance in *N. parvum* [32]. Taking into account the number of genes encoding proteins, we finally chose to perform a gene knockout on the ABC2 gene to verify its functions.

### 2.4. Functional Validation of ABC Transporter Protein Genes

#### 2.4.1. Determination of ABC Transporter Gene Expression by Quantitative Real-Time PCR (qRT-PCR)

**Figure 9:**
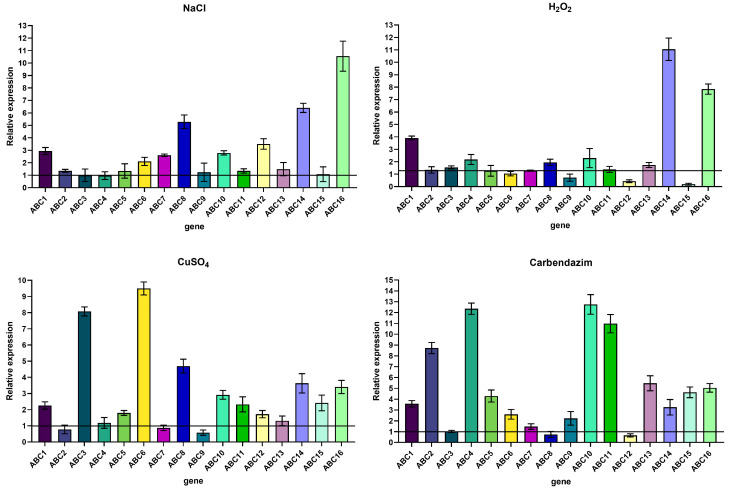
Relative expression of 16 ABC transporters genes.

The relative expression of the ABC transporter protein genes (code: *NpABC1*–*16*) of *N. parvum* strains cultured in PDA medium containing NaCl, H_2_O_2_, CuSO_4_, and polymyxin for 3 days was measured separately from that of the strain cultured in pure PDA; the results are shown in Figure 9. In the samples cultured with NaCl, the genes showing up-regulated expression were *ABC1, ABC2, ABC5, ABC6, ABC7, ABC8, ABC9, ABC10, ABC11, ABC12, ABC13, ABC14,* and *ABC16*. In the samples cultured with H_2_O_2_, the genes showing up-regulated expression were *ABC1, ABC3, ABC4, ABC8, ABC10, ABC11, ABC13, ABC14,* and *ABC16*. In the samples cultured with CuSO4, the genes showing up-regulated expression were *ABC1*, *ABC3*, *ABC4*, *ABC5*, *ABC6*, *ABC8, ABC10, ABC11, ABC12, ABC13, ABC14, ABC15,* and *ABC16*. In the samples cultured with carbendazim, the genes showing up-regulated expression were *ABC1, ABC2, ABC4, ABC5, ABC6, ABC7, ABC9, ABC10, ABC11, ABC13, ABC15,* and *ABC16*.

#### 2.4.2. Analysis of ABC2 Gene Structure

**Figure 10:**
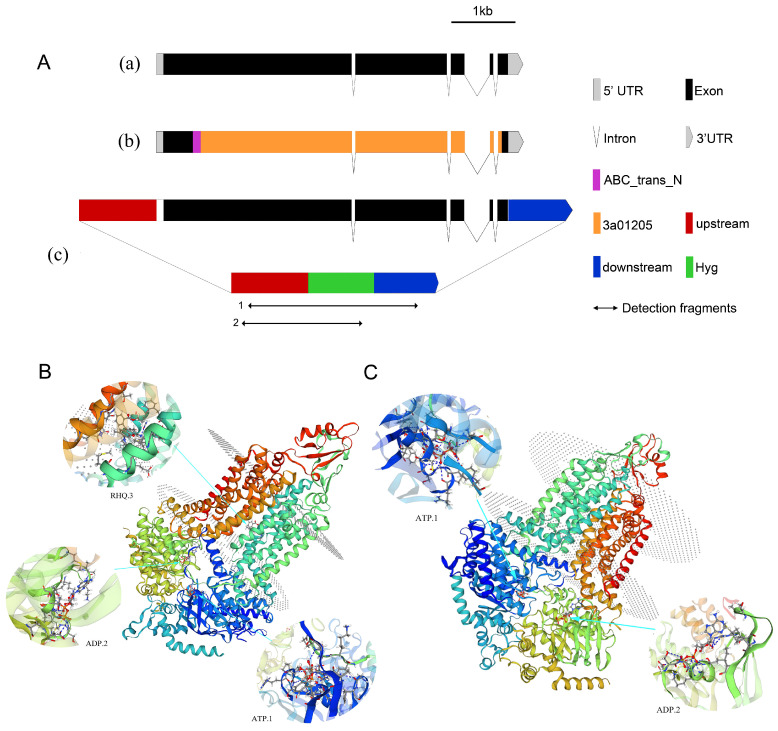
Schematic structure and homology modeling of *NpABC2* encoding proteins. (**A**): (**a**) Exon and intron distribution, (**b**) main domain distribution, (**c**) schematic diagram showing *NpABC2* gene knockout. (**B**): Homology modeling of pro9. (**C**): Homology modeling of pro18.

The *NpABC2* gene was annotated to pro9 and pro18, and its upstream and downstream 2000-bp fragments were obtained from the National Center for Biotechnology Information (NCBI) database. Analysis revealed that the gene is 5379 bp in length, encodes 1597 amino acids, possesses five exons (Figure 10A(a)), and contains an ABC_trans_N (155–232 site) and 3a01205 region (196–1563 site) (Figure 10A(b)) which are found at the N-terminus of ABC-transporter proteins in fungi, plants, and higher eukaryotes, respectively. We identified an extracellular domain and a signature structure of the pleiotropic drug resistance (PDR) family protein; the amino acid sequence was analyzed in the NCBI database with the fungal model species BLASTp, and the *Saccharomyces cerevisiae SNQ2* homolog sequence (AAB33744.1) showed the highest similarity (42.08%), followed by the brefeldin A efflux transporter *Bfr1* gene (NP_587932.3) with 41.79% similarity. The proteins encoded by the *NpABC2* gene were modeled by SWISS-MODEL; comparison results showed that the proteins that matched most frequently in the database were 7p04.1.A (Figure 10B) and 7p05.1.A (Figure 10C); these were annotated as pleiotropic ABC efflux transporters of multiple drugs.

#### 2.4.3. Knockout of the ABC2 Gene

**Figure 11:**
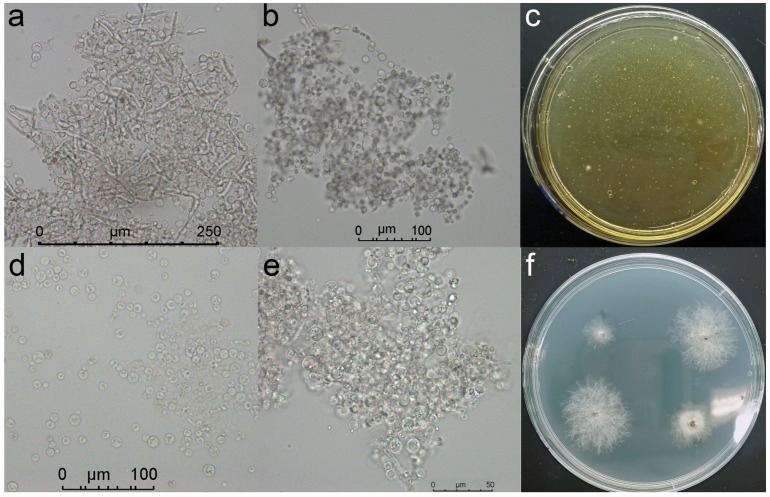
Protoplasts and transformers. (**a**) Gradual enzymatic digestion of mycelium; (**b**) enzymatic digestion of all hypha; (**c**) transformed strains were grown after 3 days of transformation culture; (**d**) protoplasts after centrifugal washing; (**e**) resuscitated protoplasts; (**f**) transformant strains after 5 days of growth.

**Figure 12:**
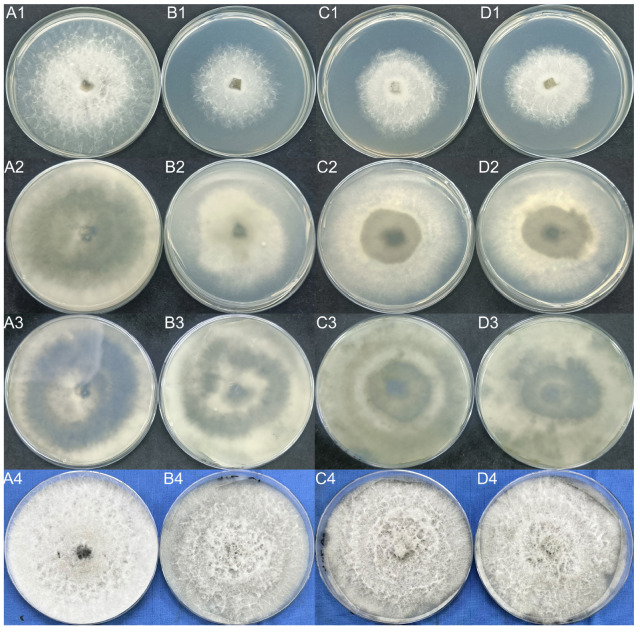
Wild-type and transformant strains were cultured in PDA medium for 2 days (**A1**–**D1**), 3 days (**A2**–**D2**), and 5 days (**A3**–**D3**, **A4**–**D4**).

DNA fragments of the upstream and downstream homologous arms of the *NpABC2* gene and the thaumatin transferase gene were obtained from the gDNA of *N. parvum* (Figure S4). The purified and recovered sequencing results were consistent with the expected sequences. The fusion fragments, upstream-hygromycin B phosphotransferase gene (Hph) and Hph-downstream, were obtained by overlap (Figure S5), and the purification and recovery sequencing results were consistent with the expected sequences. The fusion fragments were ligated into the cloning vector pClone007 separately to generate a knockout vector for the *NpABC2* gene. After incubating the fusion fragment and protoplasts for 10 h for genetic transformation, the protoplasts recovered and regrew their cell walls. Single colonies of white knockout mutants grew on plates after 3 days of incubation (Figure 11c). The electrophoresis results of colony PCR assays of the transformant strains using specific primers are shown in Figure S6. At this point, we successfully obtained a knockout transformant strain of the *NpABC2* gene (Figure 12).

#### 2.4.4. Validation of the ABC2 Knockout Mutant

**Figure 13:**
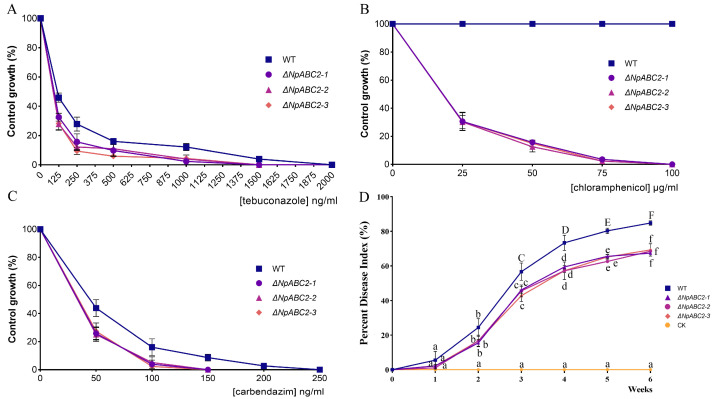
Effect of ABC2 on antifungal drugs and dynamic changes in the disease index of walnut infected by different strains. (**A**): Tebuconazole; (**B**): Chloramphenicol; (**C**): Carbendazim; (**D**): The disease index of walnut infected by different strains. WT, *ΔNpABC2-1*, *ΔNpABC2-2*, *ΔNpABC2-3,* and CK represent the changes in the disease index of walnut inoculated with wild-type, *ΔNpABC2-1*, *ΔNpABC2-2*, *ΔNpABC2-3,* and sterile deionized water, respectively.

Measurements of the knockout mutant and wild-type strains showed that the colony growth rate of the knockout transformants was significantly lower than that of the wild-type. After 2 days of culture, the colony diameter of the transformant strains was only 5.05 cm on average; this was only 59.41% of the colony diameter of the wild-type strain (Figure 11A1–D1). Furthermore, the transformant strain produced less of the black pigment (Figure 12A2–D3). After 5 days, the mycelium of the knockout mutant appeared grayer than the wild-type (Figure 12A4–D4). Furthermore, the knockout mutant was significantly more susceptible to antifungal drugs (Figure 13A–C). We compared the virulence of the knockout mutant with that of the wild-type strain and found that the virulence of the knockout mutant was significantly lower (Figure 13D).

## 3. Discussion

The ABC transporter superfamily are classified into nine subfamilies (ABCA–I). Of these, the ABCI subfamily is the newest of the prokaryote transporter subfamily and contains ABC proteins without NBDs; however, this subfamily is only found in plant taxa [35,36]. The ABCA subfamily of fungal ABC transporters are FT; they have completely lost their HT; furthermore, this subfamily has not been the focus of dedicated research. Members of the ABCA subfamily have not been detected in the genomes of many fungi. The human subfamily of ABCA transporters is currently known to be involved in the transport and metabolism of lipids [37]. In the present study, group V contains only pro21; we consider this to belong to the ABCA subfamily because it contains an ABC_subfamily_A domain. This is an ATP-binding cassette domain of the lipid transporters. A characteristic feature of the ABCA transporter is a large extracellular loop between the first two transmembrane helices in the TMD [38]; this is evident in the tertiary structure of pro21 (Figure 7).

The ABCB subfamily, the largest subfamily, is currently one of the most studied ABC transporters and performs a variety of functions, including multidrug resistance, antigen processing, the export of mitochondrial peptides and pheromones, the biogenesis of Fe/S cluster proteins, and heavy metal resistance. These were initially investigated because of their multidrug resistance [39,40,41,42,43]. In the present study, group Ⅷ contains only pro14; we consider this to belong to ABCB subfamily because it contains ATM1 and ABC_6TM_HMT1 domains. These are involved in transition metal homeostasis and detoxification processes and are also involved in the assembly of cytosolic iron-sulfur (Fe/S) cluster-containing proteins by mediating the export of Fe/S cluster precursors from mitochondria. This finding is consistent with the fact that the relative expression levels of the *ABC6* gene were up-regulated in the medium supplemented with metal, thus further illustrating the function of pro14 on drug extrusion; however, the physiological significance of ABCB transporters is not limited to drug extrusion and pheromone export alone. In *M. grisea*, ABC3 is involved in the oxidative stress response and is required for host penetration [17]. ABCB transporters with known functionality are mostly associated with multidrug resistance, although the functionality of half of the ABCB transporters is not linked to multidrug resistance; these appear to localize either to the mitochondria or the vacuolar membrane [44].

Members of the ABCC subfamily are all FT, most of which possess an extension of the N-terminal hydrophobic transmembrane structural domain and are known as multi-drug resistance proteins (MRP) or cystic fibrosis transmembrane conductance regulators (CFTR) [45]. The substrates of the ABCC subfamily are mainly organic anions and drugs [46,47,48]. In the present study, group Ⅶ contains four proteins; we consider these to belong to the ABCC subfamily. All four proteins possess the ABC_6TM_MRP domain. The MRP subfamily (ABCC subfamily) is composed of 13 members; of these, MRP1 to MRP9 are the major transporters that cause multidrug resistance in tumor cells by pumping anticancer drugs out of the cell. These nine MRP members function as ATP-dependent exporters of endogenous substances and xenobiotics. The MRP family can be divided into two groups depending on their structural architecture. MRP4, MRP5, MRP8, and MRP9 (ABCC4, 5, 11, and 12, respectively) possess a typical ABC transporter structure and are composed of two transmembrane domains (TMD1 and TMD2) and two nucleotide domains (NBD1 and NBD2). Conversely, MRP1, 2, 3, 6, and 7 (ABCC1, 2, 3, 6, and 7, respectively) have an additional five N-terminal transmembrane segments in a single domain (TMD0) connected to the core (TMD-NBD) by a cytoplasmic linker (L0). These four proteins are encoded by *ABC5*, *ABC10*, *ABC11,* and *ABC15,* respectively; their expression levels in medium supplemented with carbendazim were up-regulated. A recent study in *Candida albicans* shows the role of a vacuolar ABC transporter in the sequestration of an azole drug in vacuole [49]. Three proteins in this group localized to the vacuolar membrane, thus suggesting that the multidrug resistance functionality of some ABCC transporters is to utilize ABC transporters to sequester drugs into the vacuole for storage and thereby provide drug resistance. The other protein localizes to the cell membrane and may be involved in drug efflux.

The ABCD subfamily is found in most fungi and is mainly localized in the peroxisomes [2,50,51]. Corresponding mutants are unable to grow on fatty acids such as palmitate or oleate as a sole carbon source [52]. In the present study, group Ⅳ contained three proteins, two of which localize to peroxisomes and contain the ABCD_peroxisomal_ALDP domain; this is an ATP-binding cassette domain of the peroxisomal transporter. The peroxisomal ATP-binding cassette transporter (Pat) is involved in the import of very long-chain fatty acids (VLCFA) into the peroxisome. The peroxisomal membrane forms a permeability barrier for a wide variety of metabolites required for and formed during fatty acid beta-oxidation. To communicate with the cytoplasm and mitochondria, peroxisomes need dedicated proteins to transport hydrophilic molecules across their membranes. The other protein localizes to the mitochondria and contains an ADCK1 domain. Eukaryotes contain at least three ABC1-like proteins; of these, yeast Abc1p and its human homolog ADCK3 are atypical protein kinases that are required for the biosynthesis of coenzyme Q (ubiquinone or Q), an essential lipid component in respiratory electron and proton transport. ABC1 kinases are not related to the ATP-binding cassette (ABC) membrane transporter family; thus, the database yielded an inaccurate annotation for pro17, as with pro1 (group Ⅰ). We consider that pro11 and pro13 belong to the ABCD subfamily. The relative expression levels of ABC14 (encoded protein 13) and ABC16 (encoded protein 11) were up-regulated in the medium supplemented with H_2_O_2_, as expected.

The ABCE and ABCF subfamilies possess only the NBD and are not known to have a transport function [53]; groups Ⅲ and Ⅵ concurred with this categorization. In the present study, group III featured pro7 and pro24, which both possessed the AfuA domain. This belongs to the ABC-type Fe^3+^ transport system and the periplasmic component and is involved in inorganic ion transport and metabolism. These proteins may belong to the ABCE subfamily; their subcellular localization is unknown. Group Ⅵ featured four proteins; these had no transmembrane structure and were localized in the mitochondria or cytoplasm. These proteins possessed a number of domains, including PLN03073, ABCF_EF_3, Znuc, FecE, NAta, FepC, EcfA2, and TauA. All of these are associated with inorganic ion transport and metabolism, including Fe^3+^, Mn^2+^, Na^+^, K^+^, and Zn^2+^. We consider these proteins as the ABCF subfamily. The expression levels of protein coding genes for these two subfamilies were up-regulated in medium supplemented with metal ions and sodium chloride. With the results of pro22 in PPI network analysis, we suggest that this family may involve in oxidative stress and drug transport.

All of the ABC transporters in the fungal ABCG subfamily are associated with multi-drug resistance and are known as PDR transporters; these play a role in the multi-drug resistance of fungi [54,55,56]. The characteristic feature distinguishing ABCG transporters from other subfamilies is their reverse topology; in other words, the nucleotide-binding domain precedes the transmembrane domain [44,57]. In the present study, group Ⅲ is the ABCG subfamily and contains eight proteins; these all possess ABCG_PDR and 3a01205 domains. The possession of these two domains prove that these proteins belong to the ABCG subfamily. Five *S. cerevisiae* transporters (Snq2p, Pdr5p, Pdr10p, Pdr11p, and Pdr15p) are part of the PDR network and contribute to pleiotropic drug resistance by exporting various, chemically unrelated hydrophobic molecules from the cell [44,58]. The function of PDR proteins is not limited to drug export, as *Pdr11p* and *Aus1p* are required for sterol uptake and anaerobic growth [44,59]. In addition, a role for two PDR proteins (Snq2p and Pdr5p) has recently been proposed in yeast quorum sensing [44,60]. From the results of PPI network analysis, we can infer that the ABCG family is also directly associated with oxidative stress and ion transport, and indirectly affects sugar transport. In fact, the expression levels of the genes encoding these proteins were up-regulated in media supplemented with carbendazim, sodium chloride, and hydrogen peroxide. Furthermore, two proteins (pro9 and pro18)—encoded by the *ABC2* gene that we knocked out—both belong to the ABCG subfamily. Our knockout transformant strains did appear to be more sensitive to several different antifungal agents. This also allowed us to confirm that the *ABC2* gene plays a role in multidrug resistance in *N. parvum* and, furthermore, that its deletion also leads to the attenuation of vegetative growth and virulence.

In conclusion, *NpABC2* is involved in vegetative growth and plays a role in multidrug resistance. It also helps *N. parvum* cope with the toxic environment present in the host during infection. However, the specific function of the *ABC2* gene in the vegetative growth of *N. parvum* remains unknown. Furthermore, the functionality of the other ABC transporters remains to be explored and validated in the future.

## 4. Materials and Methods

### 4.1. Reagents, Equipment and Experimental Materials

*N. parvum* was isolated by the tissue isolation method [61] from branch blight disease of walnut. The accession numbers of the *ITS*, *tef1*, and *TUB2* genes for strain BH01 in the NCBI database are OM980641, ON000394, and ON000396; the accession numbers of the *ITS*, *tef1*, and *TUB2* genes for strain BH03 in the NCBI database are OM980642, ON000395, and ON000397, respectively. The plant tissue samples were collected from five-year-old walnut branch blight samples in Ya′an city (102°59′ N, 29°58′ E) at an altitude of 738.35 m with an annual temperature of 6.1–25.3 °C and an annual precipitation of 1204–2367 mm. The pathogenic test plants were purchased from Sichuan Chengdu Fujiao Seeding Co., Ltd., China, and planted in the greenhouse of Sichuan Agricultural University in Chengdu city (30°42′ N, 103°51′ E).

### 4.2. Methods

#### 4.2.1. Biological Material

We combined the methods of Andrade et al. [62] with those of Morales-Cruz et al. [63] to perform transcriptome profiling of the fungal cultures on solid medium. Two strains of *N. parvum* were inoculated onto PDA coated with 2 mL of sterile water to collect SK1, SK2, and SK3 (three biological replications of strain BH01) and WK1, WK2, and WK3 (three biological replications of strain BH03). In addition, two strains were inoculated on SuperPure agar containing fine ground walnut wood coated with 2 mL of sterile walnut extract to collect SW1, SW2, and SW3 (three biological replications of strain BH01) and WW1, WW2, and WW3 (three biological replications of strain BH03). Cultures were incubated at 25°C for 7 days, sub-cultured into the same medium, and incubated again for 7 days. Sub-culturing was carried out three times before the protein extraction of mycelium harvested from the surface of the agar.

#### 4.2.2. Total Protein Extraction and Quality Testing

The 12 samples were ground individually in liquid nitrogen and lysed with lysis buffer containing 100 mM NH_4_HCO_3_ (pH = 8), 8 M urea, and 0.2% SDS, followed by 5 min of ultrasonication on ice. The lysate was centrifuged at 12,000× *g* for 15 min at 4 °C, and the supernatant was transferred to a clean tube. Extracts from each sample were reduced with 10 mM DTT for 1 h at 56 °C and subsequently alkylated with sufficient iodoacetamide for 1 h at room temperature in the dark. Then, samples were completely mixed with 4 volumes of precooled acetone by vortexing and incubated at −20 °C for at least 2 h. Samples were then centrifuged, and the precipitation was collected. After washing twice with cold acetone, the pellet was dissolved by dissolution buffer containing 0.1 M triethylammonium bicarbonate (TEAB, pH = 8.5) and 6 M urea.

BSA standard protein solution was prepared according to the instructions of the Bradford Protein Quantitative Kit (Beyotime, Shanghai, China), with concentrations ranging from 0 to 0.5 g/L; this allowed us to determine the protein concentrations of individual samples.

#### 4.2.3. TMT Labeling of Peptides

First, 120 μg of each protein sample was taken and the volume was made up to 100 μL with dissolution buffer; then, 1.5 μg trypsin and 500 μL of 100 mM TEAB buffer were added. The sample was mixed and digested at 37 °C for 4 h. Next, 1.5 μg trypsin and CaCl_2_ were added, and the sample was digested overnight. Formic acid was mixed with the digested sample, the pH adjusted to <3, and the sample centrifuged at 12,000× *g* for 5 min at room temperature. The supernatant was slowly loaded onto a C18 desalting column, washed with washing buffer three times, then eluted by elution buffer (0.1% formic acid, 70% acetonitrile). The eluents of each sample were collected and lyophilized. Next, 100 μL of 0.1 M TEAB buffer was added to reconstitute the sample and 41 μL of acetonitrile-dissolved TMT labeling reagent was added. The sample was then mixed by shaking for 2 h at room temperature. Then, the reaction was stopped by adding 8% ammonia. All labeling samples were mixed in an equal volume, desalted, and lyophilized.

#### 4.2.4. Separation of Fractions and Liquid Chromatography Tandem Mass Spectrometry (LC-MS/MS) Analysis

Mobile phase A and B were used to develop a gradient elution. The lyophilized powder was dissolved in solution A and centrifuged at 12,000× *g* for 10 min at room temperature. The sample was then fractionated using a C18 column on a Rigol L3000 HPLC system; the column oven temperature was set to 50 °C. Details of the elution gradient are given in Table 1. The eluates were monitored at UV 214 nm, collected in one tube per minute, and combined into 10 fractions. All fractions were dried under vacuum and reconstituted in 0.1% formic acid in water. To construct the transition library, shotgun proteomics analyses were performed using an EASY-nLCTM 1200 UHPLC system coupled with an Q Exactive HF-X mass spectrometer operating in the data-dependent acquisition (DDA) mode. Then, 1 μg of sample was injected into a home-made C18 Nano-Trap column. Peptides were separated in a home-made analytical column using a linear gradient elution. The separated peptides were analyzed by a Q Exactive HF-X mass spectrometer with an ion source of Nanospray Flex, a spray voltage of 2.3 kV, and an ion transport capillary temperature of 320 °C. We used a full scan range from m/z 350 to 1500 with a resolution of 60,000. The automatic gain control target value was 3 × 10^6^ and the maximum ion injection time was 20 ms. The top 40 precursors of the highest abundance in the full scan were selected and fragmented by higher energy collisional dissociation and analyzed by MS/MS, in which the resolution was 45,000 for 10 plex, the automatic gain control target value was 5 × 10^4^, the maximum ion injection time was 86 ms, the normalized collision energy was set to 32%, the intensity threshold was 1.2 × 10^5^, and the dynamic exclusion parameter was 20 s.

#### 4.2.5. Identification and Quantitation of Protein

A protein database search was performed after mass spectrometry to identify protein sequences. We used Proteome Discoverer 2.4 to search the NCBI 463891-X101SC19061477-Z01-Neofusicoccum_parvum-NCBI.fasta resource (10,366 sequences); the search parameters are shown in Table 1. To improve the quality of the results and reduce the false positive rate, Proteome Discoverer software further filtered the search results; peptide spectrum matches (PSMs) with a confidence level of 99% or higher were considered as plausible PSMs. Proteins containing at least one unique peptide (unique peptide) were considered as plausible proteins. We only retained the peptides and proteins that were plausible and performed FDR validation to remove the peptides and proteins with an FDR > 1%. We also performed a series of quality control tests, including peptide length distribution, parent ion mass tolerance distribution, unique peptide number distribution, protein coverage distribution, and protein molecular weight distribution. The protein quantitation results were statistically analyzed with the t-test. Proteins for which the quantitation differed significantly between experimental and control groups, (*p* < 0.05 and |log2FC| > 1.5 or |log2FC| < 0.67) were defined as DEPs.

#### 4.2.6. Functional Analysis of Protein

GO and IPR functional analysis were conducted using the InterProScan program against the non-redundant protein database (including Pfam, PRINTS, ProDom, SMART, ProSite, PANTHER) [64]. In addition, the COG and KEGG databases were used to analyze protein families and pathways.

#### 4.2.7. GO and KEGG Enrichment Analysis of DEPs

To identify the biological processes that were significantly associated with differential proteins and biological functions, we performed enrichment analysis for the differential genes. GO functional significant enrichment analysis identified the GO functional entries that were significantly enriched for the differential proteins when compared to all identified protein backgrounds. All differential proteins were first mapped to each term in the Gene Ontology database (http://www.geneontology.org/, accessed on 18 September 2020). The number of proteins per term was calculated, and then hypergeometric tests were applied to identify GO entries that were significantly enriched in differential proteins when compared to all protein backgrounds. The *p*-value was calculated and the GO terms satisfying this condition were defined as the GO terms that were significantly enriched in the differential proteins using a threshold value of *p* ≤ 0.05. The GO significance analysis enabled us to identify the main biological functions exercised by differentially expressed proteins. DEPs were used for volcanic map analysis, cluster heat map analysis, and enrichment analysis of GO, IPR, and KEGG terms [65].

#### 4.2.8. Subcellular Localization Analysis of Differentially Expressed Proteins

The eukaryotic Cell-mPLOC 2.0 database was used to annotate the subcellular localization of differentially expressed proteins. The cells of an organism represent a highly ordered structure and intracellular localization can be divided into different organelles or cellular regions depending on spatial distribution and function, including the nucleus, Golgi apparatus, endoplasmic reticulum, mitochondria, cytoplasm, and cell membrane.

#### 4.2.9. Phylogenetic, Structural, and Functional Analysis of ABC Transporter Proteins in the Proteome

Using protein sequences, we next performed a phylogenetic analysis. The protein sequences were aligned with ClustalW using MEGA version 7.0.26 (Tamura K, Stecher G & Kumar S., State College, PA, USA) with default parameters [66]. A phylogenetic tree was constructed using the maximum likelihood method [67] in MEGA7.0 software with 1000 bootstrap replicates. 

Motifs of the ABC transporter were obtained from the MEME database (https://meme-suite.org/meme/tools/meme, accessed on 5 January 2021). Domains were obtained from the NCBI database (https://www.ncbi.nlm.nih.gov/Structure/cdd/wrpsb.cgi, accessed on 5 January 2022). The prediction of physicochemical properties was performed with ProtParam (https://web.expasy.org/protparam/, accessed on 5 January 2021). Hydrophilicity/hydrophobicity prediction was performed with ProtScale (https://web.expasy.org/protscale/, accessed on 8 January 2021). Protein signal peptide prediction was obtained from SignalP 4.1 (http://www.cbs.dtu.dk/services/SignalP/, accessed on 8 January 2021). Protein secondary structure prediction was performed with SOPMA (https://npsa-prabi.ibcp.fr/cgi-bin/npsa_automat.pl?page=npsa_sopma.html, accessed on 10 January 2021). Protein tertiary structure prediction was performed with SWISS-MODEL (https://swissmodel.expasy.org/interactive, accessed on 15 January 2021).

#### 4.2.10. Analysis of PPI Network in ABC Transporters

The probable protein-protein interactions were predicted using the STRING-db server [68] (http://string.embl.de/, accessed on 20 January 2021). In addition, *Neofusicoccum parvum* data were extracted directly from the database to derive corresponding interaction information, and Cytoscape version 3.9.1 (Shannon P, Markiel A, Ozier O, Baliga NS, Wang JT, Ramage D, Amin N, Schwikowski B & Ideker T. Seattle, WA, USA) was used to construct the network diagram from which information on ABC transporters and their first neighbor was extracted.

#### 4.2.11. The Determination of Expression Levels by qRT-PCR

We inoculated the wild-type strain on PDA plates (containing 1.2M NaCl, 60mM H_2_O_2_, 1.5 mM CuSO4, and 50 ng/mL carbendazim, pure PDA as the control) and collected mycelium after 3 days. Then, total RNA was extracted with TransZol Up (TransGen Biotech Co., Ltd., Beijing, China) in accordance with the manufacturer′s instructions. Primer3Plus (http://primer3plus.com/cgi-bin/dev/primer3plus.cgi, accessed on 2 February 2021) was used to design primers for the 17 *ABC* genes and the reference gene glyceraldehyde-3-phosphate dehydrogenase *(GAPDH*) (Table S2). The CFX96 Real-Time PCR Detection System (BIO-RAD Laboratories, Inc., Hercules, CA, USA) was used to quantify changes in the expression levels of the 17 genes. cDNA was obtained using the PrimeScript RT Reagent Kit with gDNA Eraser (TaKaRa Biomedical Technology (Beijing) Co., Ltd., Beijing, China) in accordance with the manufacturer′s recommendations. The qPCR reactions were performed with TB Green Fast qPCR Mix (TaKaRa) in accordance with the manufacturer’s recommendations. Each reaction was repeated three times, and the mean value was calculated. *GAPDH* was used as an internal reference gene to detect the expression changes in the different treatment groups. Data were analyzed by the 2^−ΔΔCt^ method [69].

#### 4.2.12. Knockout Plasmid Construction

The *Hph* gene was amplified from pSilent-1 plasmid DNA as a screening marker gene. Taking the ATP-binding cassette transporter 2 (ABC2) gene as the center, we selected 1100–1500 bp regions upstream and downstream as homologous arms to design primers (Table S3) to amplify homologous arms of ABC1 from *N. parvum* gDNA. The gDNA of *N. parvum* was extracted by Column Fungal DNAout 2.0 (TIANDZ, Inc., Beijing, China). Amplification system: 1 µL of DNA, 1 µL of the forward and reverse primers, 25 µL of 2× TransTaq HiFi PCR SuperMix (TaKaRa), and 22 µL of ddH_2_O. The reaction procedure was as follows: 94 °C for 5 min, followed by 35 cycles of 94 °C for 30 s, 55 °C for 30 s, and 72 °C for 1 min 30 s, and a final step of 72 °C for 8 min. Upstream/downstream and *Hph* gene fragments were obtained. The fusion fragments were obtained by overlap extension PCR [70]. The amplification system (30 µL total) contained 6 µL of upstream/downstream DNA, 6 µL of *Hph* DNA, 3 µL of 10× LA buffer, 1 µL of dNTP Mix, 0.3 µL of LA Taq (TaKaRa), and 13.7 µL of ddH_2_O. The reaction procedure was as follows: 94 °C for 5 min, followed by 35 cycles of 94 °C for 30 s, 55 °C for 1 min, and 72 °C for 2 min 30 s, and a final step of 72 °C for 8 min. The fusion fragment upstream-Hph and Hph-downstream fragments were obtained. The vector pClone007 was used to ligate the fusion fragment. The recombinant plasmid was transformed into DH5α and detected by colony PCR.

#### 4.2.13. Protoplast Preparation and PEG-Mediated Protoplast Transformation

The protoplasmic preparation of *N. parvum* and polyethylene glycol (PEG)-mediated genetic transformation were described previously [32]. Direct PCR lysis buffer for microorganisms (TaKaRa) was used for the detection of transformants (Table S3).

#### 4.2.14. Phenotypic Analysis and the Determination of Sensitivity

To investigate the morphology of colonies from the different strains and to analyze the difference in vegetative growth between strains, we inoculated the strains on PDA plates (9 × 9 cm) and measured and observed the strains after 3 and 5 days. To determine whether *NpABC2* plays a role in multidrug resistance, we tested the effects of anti-fungal drugs on wild-type and mutant strains. The tested drugs included protein synthesis inhibitors (chloramphenicol) and sterol biosynthesis inhibitors (carbendazim and tebuconazole). For all substances, stock solutions were prepared in dimethyl sulfoxide (DMSO). Mycelial plugs with unamended PDA were used as the inoculum to determine the effect of the compounds on vegetative growth. At least three plates were used for each compound and concentration.

#### 4.2.15. Pathogenicity Testing

Three-year-old, healthy *J. regia* with a uniform growth status were used for pathogenicity testing. Each group of 10 plants was selected randomly. Branches with uniform growth from the top three branches of each plant were selected and covered with mycelial plugs from the wild-type strain, three mutant strains, and pure PDA. The samples were bagged to retain moisture and sprayed with sterile water once every 24 h. The incidence of disease was investigated 20 days after inoculation. The disease severity was assessed regularly using a 0–5 scale: 0 = no infection, 1 = 1–20% infection, 2 = 21–40% infection, 3 = 41–60% infection, 4 = 61–80% infection, and 5 = more than 80% infection [16]. The percent disease index (*PDI*) was determined following the standard formula described previously [71]:

$$PDI=\frac{\sum \left(Ni\times Di\right)}{N\times Dm}\times 100$$

*D_i_*: disease grade, *N_i_*: number of diseased branches, *N*: total number of branches, *D_m_*: the most serious disease grade.

## Figures and Tables

**Table 1:** The parameters used for Proteome Discoverer analysis.

Item	Value
Type of Quantification	Reporter Quantification (TMT)
Enzyme	Trypsin
Max/Missed Cleavage Sites	2
Precursor Mass Tolerance	10 ppm
Fragment Mass Tolerance	0.02 Da
Dynamic Modification	Oxidation/+15.995 Da (M) and TMT/+229.163 Da (K,Y)
N-Terminal Modification	Acetyl/+42.011 Da (N-Terminal) and Met-loss/−131.040Da(M) and Met-loss+Acetyl/−89.030 Da(M)
Static Modification	Carbamidomethyl/+57.021 Da (C)

Note: (1) type of quantification: type of quantitative method; (2) enzyme: type of enzyme cleavage; (3) max/missed cleavage sites: maximum number of enzyme missed sites allowed; (4) Precursor mass tolerance: mass deviation tolerance range during library searching by precursor ions; (5) fragment mass tolerance: tolerance range of quality deviation when searching for fragment ions; (6) dynamic modification: set specific variable modification type; (7) N-terminal modification: modification type of N-terminal; (8) static modification: set a specific fixed modification type.

## Data Availability

Not applicable.

## Supplementary Materials

The following supporting information can be downloaded at: https://www.mdpi.com/article/10.3390/ijms23179908/s1.
