# Supplementary material for: Tandem Mass Tags Quantitative Proteome Identification and Function Analysis of ABC Transporters in Neofusicoccum parvum
Source: Int J Mol Sci. 2022 Aug 31;23(17):9908. doi: 10.3390/ijms23179908 (PMC9456026; doi:10.3390/ijms23179908)
Supplement: Supplementary file 1 [file ijms-23-09908-s001.zip › ijms-1861981-supplementary.pdf]

Table S1. Analysis of the physicochemical properties of proteins

Protein	Number of AA	Molecular weight	Theoretical pI	Asp +	Arg + Lys	Formula	Instability index	Aliphatic index	GRAVY
pro1	1160	132555.13	6.83	156	153	$C_{5976}H_{9224}N_{1618}O_{1723}S_{39}$	36.01	76.91	-0.430
pro2	1435	159290.13	6.08	149	133	$C_{7202}H_{11096}N_{1894}O_{2080}S_{56}$	29.98	85.84	-0.068
pro3	2115	235138.26	5.7	250	217	$C_{10512}H_{16497}N_{2861}O_{3149}S_{56}$	38.54	91.12	-0.176
pro4	1676	185084.38	6.22	182	169	$C_{8313}H_{12906}N_{2238}O_{2457}S_{49}$	43.64	85.06	-0.124
pro5	1561	173055.19	6.75	170	166	$C_{7800}H_{12308}N_{2126}O_{2232}S_{46}$	40.00	101.97	0.083
pro6	1094	120407.53	5.54	124	102	$C_{5375}H_{8390}N_{1446}O_{1594}S_{51}$	41.95	89.28	-0.060
pro7	362	40357.12	4.59	45	27	$C_{1831}H_{2748}N_{468}O_{553}S_{6}$	37.29	79.53	-0.277
pro8	1524	171038.91	6.31	156	147	$C_{7685}H_{11816}N_{2044}O_{2247}S_{70}$	37.92	79.99	-0.163
pro9	1408	157128.35	6.24	170	164	$C_{7037}H_{10954}N_{1896}O_{2104}S_{42}$	34.88	80.01	-0.258
pro10	1396	154596.86	8.67	158	167	$C_{6878}H_{10993}N_{1923}O_{2015}S_{54}$	37.26	93.24	-0.115
pro11	828	92647.95	9.24	92	105	$C_{4161}H_{6542}N_{1166}O_{1196}S_{19}$	33.30	90.13	-0.248
pro12	1368	154161.17	6.58	147	141	$C_{6986}H_{10784}N_{1868}O_{1964}S_{56}$	38.70	89.07	-0.009
pro13	7005	80171.69	9.00	84	93	$C_{3614}H_{5749}N_{983}O_{1031}S_{22}$	42.21	100.26	-0.100
pro14	957	107095.73	8.63	96	103	$C_{4819}H_{7555}N_{1317}O_{1389}S_{29}$	36.64	91.49	-0.138
pro15	1342	147215.6	6.08	141	129	$C_{6599}H_{10437}N_{1761}O_{1964}S_{42}$	37.60	95.99	0.021
pro16	301	33318.72	6.02	40	35	$C_{1486}H_{2303}N_{411}O_{442}S_{10}$	41.12	76.84	-0.364
pro17	651	73798.72	9.77	69	91	$C_{3304}H_{5170}N_{952}O_{923}S_{25}$	39.61	77.96	-0.465
pro18	1374	154072.5	8.86	134	151	$C_{6975}H_{10756}N_{1848}O_{1992}S_{53}$	40.46	79.72	-0.167
pro19	285	31343.84	6.33	36	34	$C_{1398}H_{2236}N_{388}O_{417}S_{6}$	41.35	98.84	-0.255
pro20	441	49693.31	6.58	58	55	$C_{2200}H_{3467}N_{635}O_{656}S_{12}$	55.12	82.54	-0.514
pro21	1521	165315.43	6.29	133	123	$C_{7450}H_{11779}N_{1995}O_{2155}S_{48}$	34.57	103.92	0.229
pro22	344	37053.17	6.22	53	51	$C_{1613}H_{2669}N_{475}O_{509}S_{6}$	39.64	91.60	-0.342
pro23	1214	133872.96	6.63	124	120	$C_{5988}H_{9453}N_{1635}O_{1741}S_{52}$	48.61	94.74	0.041
pro24	352	39483.23	4.92	44	30	$C_{1800}H_{2693}N_{467}O_{530}S_{4}$	29.18	77.56	-0.401

Table S2. The primers used for qRT-PCR

Gene	Description	Primer	Protein
ABC1	abc transporter protein	TGCAACAATATCGCGGCATC;	Pro4, Pro6, Pro20,
ABC2	abc drug exporter protein	AACAGCGAAAACGCTTGACC;	Pro9, Pro18
ABC3	abc-type fe3^{+} transport system protein	TGTCGAAAACCGCACTCTTG;	Pro7, Pro24
ABC4	abc transporter cdr4 protein	ATGAGTTGCTGCGGTTTGAG;	Pro8, Pro12
ABC5	abc metal ion transporter protein	AGCGTTTGGTTGAAGCAGTG;	Pro3
ABC6	vacuolar abc heavy metal transporter protein	TACGTACATGGCGCAATTGC;	Pro14
ABC7	abc1 family protein	TTTGCCCACATCATCAACGC;	Pro1
ABC8	abc protein.	GTTCTACAACAAGCTCGCCAA;	Pro19
ABC9	abc1 domain protein2	ACAACCGTTTCTTGGGATCG;	Pro17
ABC10	abc multidrug transporter protein	ATTCTCGCTGCTTGTGTTGC;	Pro10
ABC11	abc multidrug transporter mdr1 protein	TTGTCATTGCGCATCGACTG;	Pro15
ABC12	abc-type nitrate sulfonate bicarbonate transport systems	ACCGCTGACTTCTTCATGTG;	Pro16
ABC13	abc transporter-like protein	AGATCACGCACTTTGGCATC;	Pro2
ABC14	abc fatty acid protein	TGAAGAAAATGCGGCAGTGC;	Pro13
ABC15	abc bile acid protein	TTGCCTTGGCAACGATGTAC;	Pro5
ABC16	peroxisomal abc transporter protein	TTTGCCTCGTACACCAATGC;	Pro11
GAPDH	-	CTACTACATCGTCGAGTCCACC;	-

Table S3. The primers used for gene knockout and detection

Primer name	Primer sequence 5′–3′	Purpose
ABC2-up-F	CGAATGGGCTTATAGGCTTGA	Amplified homologous upstream
ABC2-up-R	agttcaggctttttcatatcGCGATTGAGTGCGGGTGT	
ABC2-down-F	cgagggcaaaggaatagagtGGGTGCTAACCGCCGAGGAA	Amplified homologous downstream
ABC2-down-R	CGAGGAGGACAAAGAAGCCCAAAC	
hyg-F	GATATGAAAAAGCCTGAACT	Amplified Hyg gene
hyg-R	ACTCTATTCCTTTGCCCTCG	
Detection fragment1-F	GGTGGCATCGATGTTGAAGC	Detection of fragment 1 from
Detection fragment1-R	ACCGAGGACGCCCTTTAAAG	deletion mutant strains
Detection fragment2-F	TGCTCGGGGTGGTAGATGTA	Detection of fragment 2 from
Detection fragment2-R	TGCTGCTCCATACAAGCCAA	deletion mutant strains

Note: the underlined sequence is the homologous arm sequence of Hph.

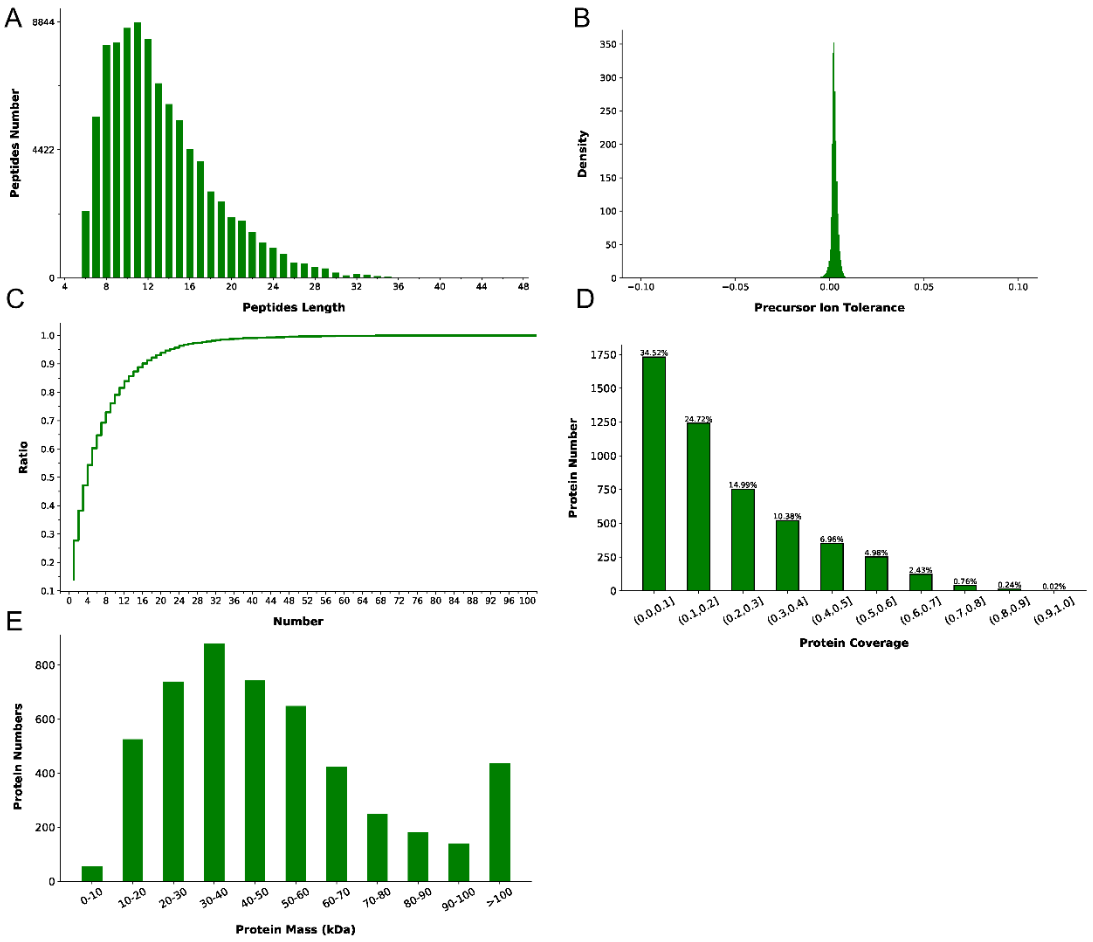

Figure S1. Results of protein control analysis. **A**: Peptide length range profiles; **B**: parent ion mass tolerance profiles; **C**: unique peptide number profiles in identified proteins; **D**: protein coverage profiles; **E**: protein molecular weight profiles.

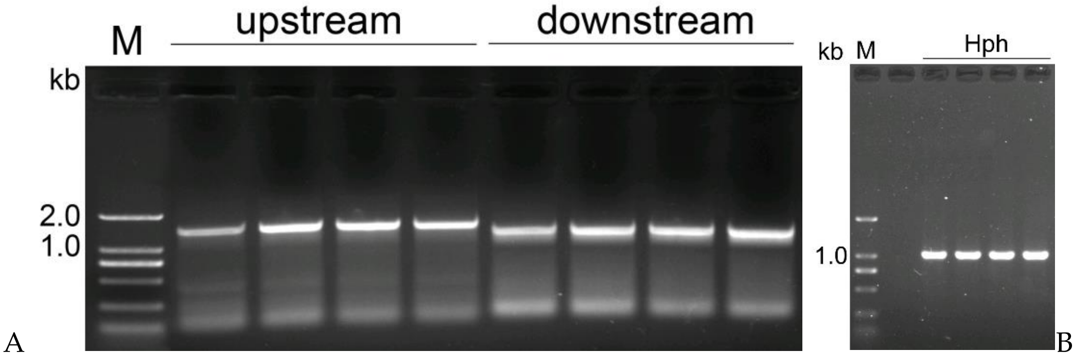

Figure S2. Agarose gel electropherograms of the PCR amplification of homology arms up- and downstream of the *NpABC2* gene from gDNA and Hph gene form pSilent-1 plasmid DNA.

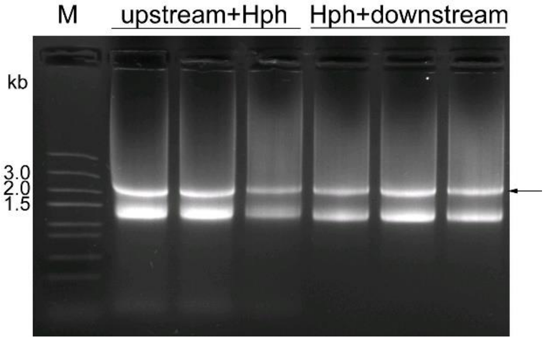

Figure S3. Agarose gel electropherograms of fusion fragments containing the up- and downstream with the Hph gene

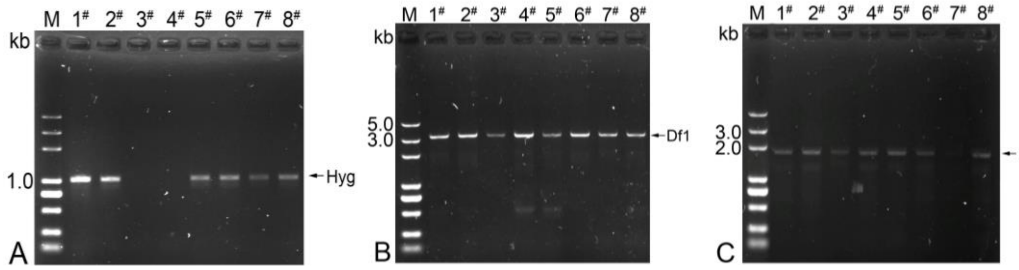

Figure S4. Agarose gel electropherogram detection by PCR of knockout transformants using detection primers. **A**: Hph primers; **B**: Detection fragment 1 primers; **C**: Detection fragment 2 primers

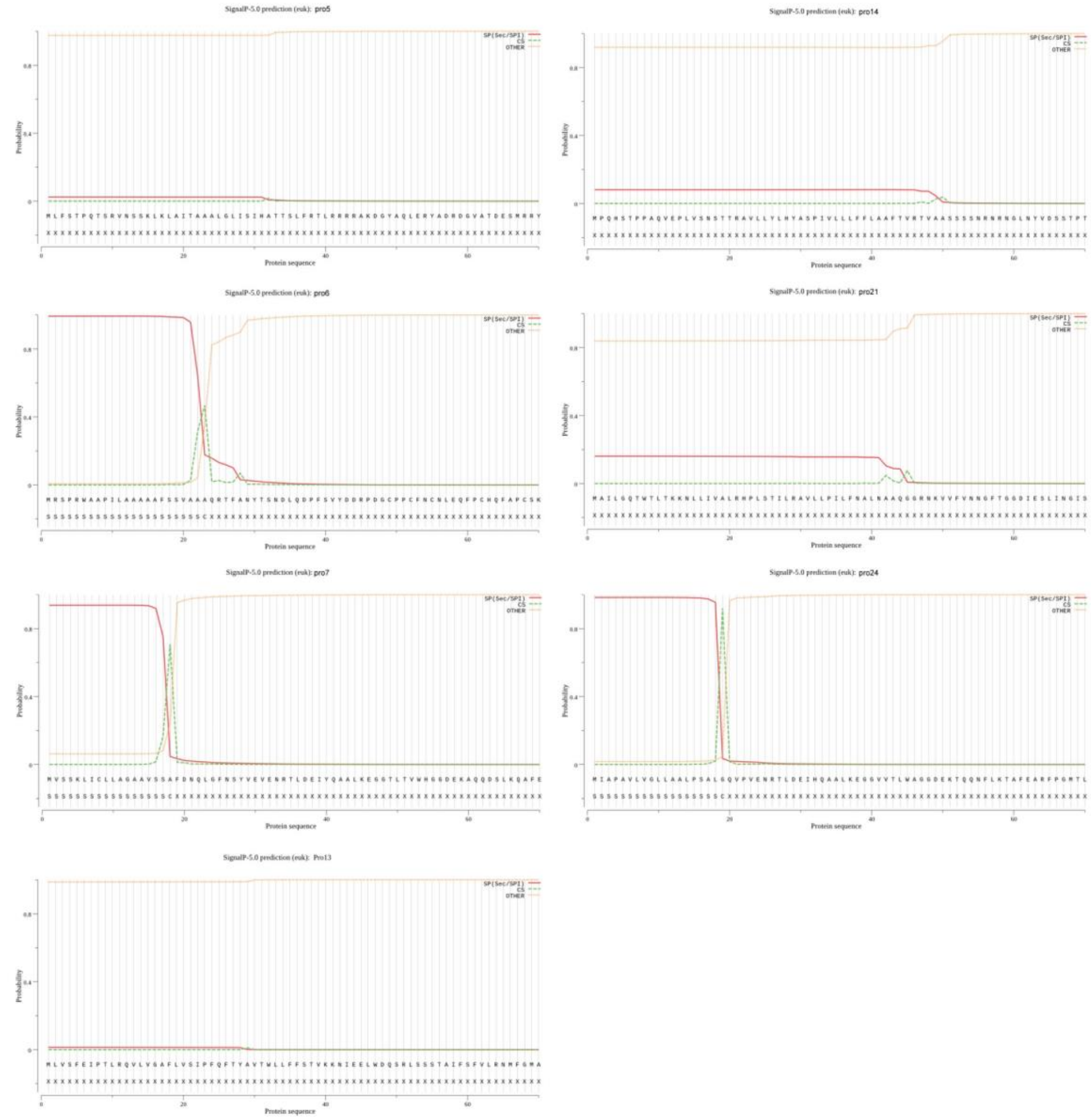

Figure S5. Signal peptide prediction maps for 24 ABC transporters

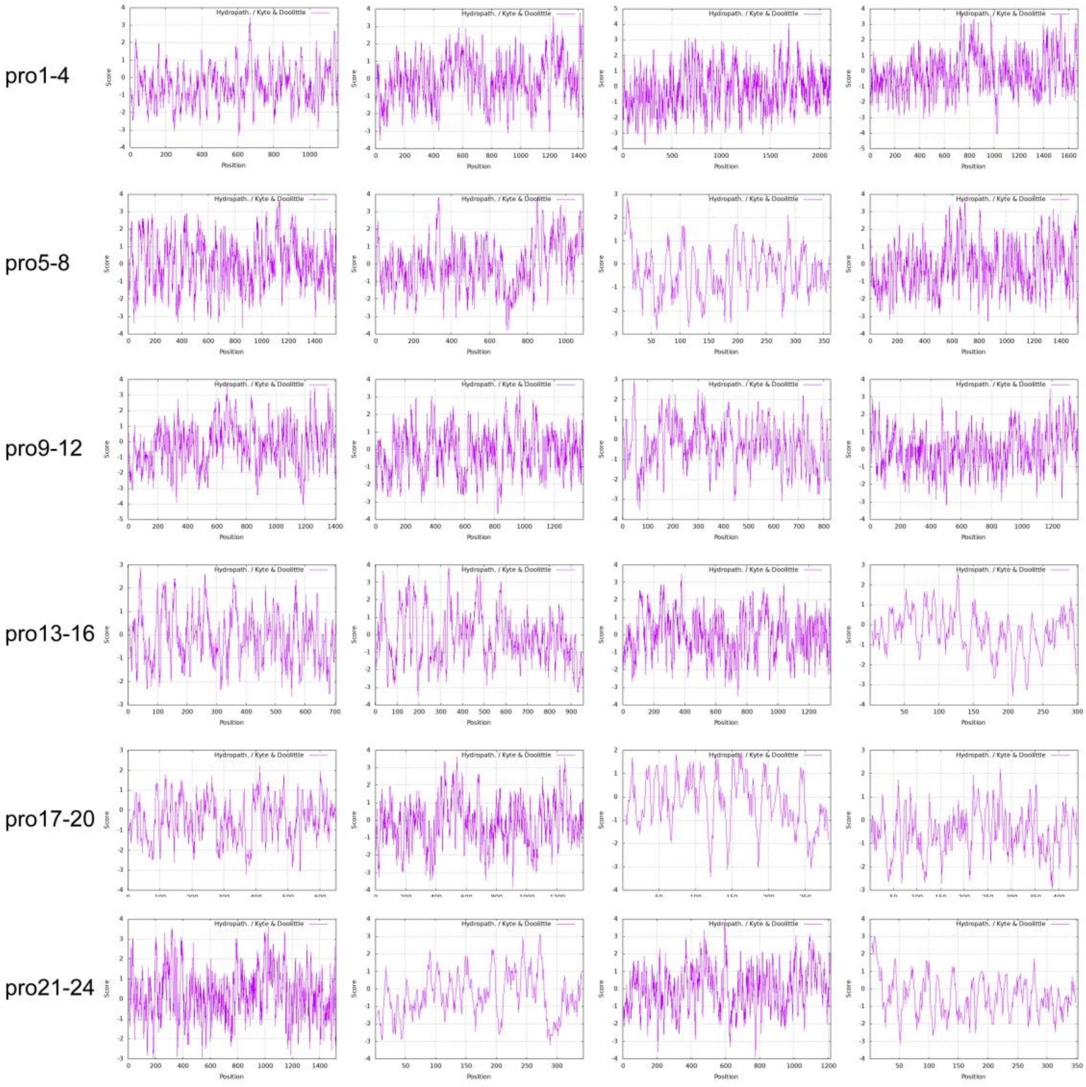

Figure S6. Hydropathy/hydrophobicity prediction chart of 24 ABC transporters
